# Corn Waste Arabinoxylans with Zinc and Thymol Nanohydroxides Coating for *Salmonella enterica* Survival on Cherry Tomato (*Solanum lycopersicum* var. *cerasiforme*)

**DOI:** 10.3390/polym17121632

**Published:** 2025-06-12

**Authors:** Jorge Manuel Silva-Jara, Ismael García-Vera, Ana María Morales-Burgos, Gabriela Hinojosa-Ventura, María Esther Macías-Rodríguez, Julia Aurora Pérez-Montaño, Zuami Villagrán, Luis Miguel Anaya-Esparza, Carlos Arnulfo Velázquez-Carriles

**Affiliations:** 1 Departamento de Farmacobiología, Universidad de Guadalajara, CUCEI, Blvd. Marcelino García Barragán 1421, Olímpica, Guadalajara 44430, Jalisco, Mexico; 2 Facultad de Ciencias Químico Biológicas, Universidad Autónoma de Sinaloa, Ciudad Universitaria, Culiacán 80013, Sinaloa, Mexico; amorales.fcqb@uas.edu.mx; 3 Departamento de Ciencias de la Salud, Centro Universitario de los Altos, Universidad de Guadalajara, Rafael Casillas Aceves 1200, Tepatitlán de Morelos 47600, Jalisco, Mexico; blanca.villagran@academicos.udg.mx; 4 Centro de Estudios para la Agricultura, la Alimentación y la Crisis Climática, Centro Universitario de los Altos, Universidad de Guadalajara, Rafael Casillas Aceves 1200, Tepatitlán de Morelos 47600, Jalisco, Mexico; 5 Departamento de Ingeniería Biológica, Sintética y de Materiales, Universidad de Guadalajara, CUTlajomulco, Carretera Tlajomulco, Santa Fé, Km 3.5, 595, Tlajomulco de Zúñiga 45641, Jalisco, Mexico

**Keywords:** corn arabinoxylan, layered hydroxide salts, edible coatings, postharvest, nanotechnology, bacterial survival, physicochemical properties

## Abstract

This research focused on the development of an edible coat made of corn waste arabinoxylan enriched with nanohybrids of zinc layered hydroxide salt and thymol (ZnHSL, ZnHSL-T). The crystallographic phase was confirmed with XRD (ICDD card 07-0155) and SEM. Filmogenic solutions prepared with the polysaccharide (AX) containing thymol (T), ZnHSL, and ZnHSL-T (AXT, AXH, and AXHT, respectively) were characterized by FTIR spectroscopy, color, thickness, transparency, and moisture content, where AXHT exhibited the thinnest layer. Furthermore, the antioxidant activity of the coatings was evaluated by the inhibition of ABTS radical, proving that thymol was present in the filmogenic solutions with inhibitions of 90%. Also, edible coatings were applied on cherry tomatoes (*Solanum lycopersicum* var. *cerasiforme*) and stored for 12 days, a period during which physicochemical properties (weight loss, color, lycopene content, soluble solids, pH, and titratable acidity) and *Salmonella* survival (serovar Enteritidis, Typhimurium, and Montevideo) were evaluated. Results demonstrated that AXHT had less weight loss than the control, and the other physicochemical properties of tomatoes were preserved. Regarding pathogen adherence, AXHT reduced the bacterial survival for *Salmonella* Enteritidis, *S.* Typhimurium, and *S.* Montevideo in 25, 30, and 45%, respectively, by day 12. The findings of this research demonstrate the application of nanotechnology to biopolymers, enabling the production of safer foods with acceptable quality parameters for consumers.

## 1. Introduction

Tomato (*Solanum lycopersicum*) is a popular fruit consumed worldwide in various culinary preparations, whether fresh or prepared. In Mexico, its cultivation represents a significant socioeconomic activity, as it contributes a substantial income and an important source of employment [1]. One of its varieties is cherry tomato (*S. lycopersicum* var. *cerasiforme*), a small, rounded fruit with high nutritional value that has increased production and consumption in recent years [2]. Cherry tomatoes have been recognized as a good source of vitamins A and C, as well as a fruit with a good taste, for which their consumption has increased [3]. Also, it can grow in diverse environments such as subtropics and semi-dry regions, it reasonably tolerates heat and drought, and it can adapt to various types of soils [3,4,5].

However, the spoilage of tomatoes has been associated with various factors, including handling, transportation, packaging, storage, sale, consumption, and the presence of bacteria such as *E. coli*, *S. aureus*, and *Salmonella* spp. [6,7]. Storage conditions significantly impact the physicochemical and sensorial properties of tomatoes, affecting parameters such as color, texture, acidity, lycopene content, and flavor, among others [8,9]. Pathogens like *Salmonella* have been recently found in tomatoes, resulting in outbreaks that affect public health and cause significant economic losses [10], particularly in developing countries. Fruits are not adequate hosts for *Salmonella*, as their acidic conditions, among other factors, prevent bacterial survival. Nonetheless, this pathogen has developed adaptability that allows it to attach to the epicarp of fruits, such as tomatoes [11,12].

Traditional methods for postharvest preservation of fruits and vegetables include thermal treatments and chemical disinfectants; however, their environmental and economic sustainability is not entirely guaranteed. For this, emerging technologies in postharvest procedures prioritize resource efficiency and reducing negative impacts on food quality while enhancing food safety and extending shelf life [13].

Edible coatings have gained attention because they can be applied to food surfaces, preventing rapid ripening due to limited gas exchange, and reducing the reproduction of deteriorative and pathogenic microorganisms, thereby extending the shelf life of food products [14,15]. In addition to functional and mechanical properties, characteristics such as color and transparency may affect the consumer’s response to a product coated with these films, considering that acceptance is highly influenced by the primary and secondary packaging of the product [16]. These coatings can be formulated from lipids, proteins, and biopolymers such as polysaccharides, in combination with additives in aqueous solutions [17]. Some biopolymers used as edible coatings include pectin, starch, chitosan, alginate, and cellulose [18,19]. Edible coatings may be prepared and dried before application to cover the food [18], while in other cases, they can be immersed in the filmogenic solution [20] and allowed to dry later. Arabinoxylan, a polysaccharide found in the cell wall of cereals, consists of a linear chain of *β*-(1-4)-linked-D-xylopyranosyl units [21] and has been applied as an edible coat on apples, improving its shelf life and reducing fruit decay [22].

Some of these biopolymeric barriers may include bioactive components, such as probiotics, antimicrobials, and antioxidant molecules, in their formulation that exert their activities at the surface level or even within the human intestinal tract [23]. Bioactive compounds, such as thymol, included in edible coatings are known for their biological properties, including antimicrobial, antioxidant, antihypertensive, immunomodulatory, and anticancer effects [24,25]. Notably, it has been demonstrated that thymol exhibits antimicrobial activity against both Gram-positive and Gram-negative bacteria, inhibiting *E. coli* and *Salmonella* Typhimurium at concentrations of 1.2 and 1.0 mmol/L, respectively [26]. This terpene has been used in packages to preserve tomatoes in refrigeration, resulting in increased lycopene and phenolic content, while reducing bacterial count associated with spoilage [27]. Nevertheless, thymol is highly thermosensitive [28] and susceptible to light exposure [29], and thus, its application in edible coating formulations remains a challenge [30]. To protect its biological properties, thymol can be encapsulated in layered compounds, which can enhance its antimicrobial and antioxidant capacities, thereby improving the inhibition of microorganisms at lower concentrations [31]. Zinc hydroxide salts also enable the controlled release of bioactive compounds, thereby prolonging their activity [32]. In this regard, nanotechnology has great potential in the food industry, whether by using sensors or disinfectants that assure innocuity and preserve the quality and nutritional content of food [33].

The present research focuses, for the first time, on the formulation of an edible coating made of arabinoxylan obtained from maize waste, enriched with nanohybrids of zinc layered hydroxide salts and thymol. It includes the characterization and evaluation of its antioxidant capacity and its application on cherry tomatoes to prevent *Salmonella enterica* adherence and survival. Tomatoes were analyzed for microbiological and physicochemical quality, considering parameters such as color, soluble solids, pH, weight loss, and titratable acidity over 12 days.

## 2. Materials and Methods

### 2.1. Raw Materials

Arabinoxylan from nixtamalized corn residues was donated by Universidad Autónoma de Sinaloa (Sinaloa, Mexico). ZnCl_2_ (Golden Bell, Guadalajara, Mexico), NaOH (Sigma Aldrich, Mexico City, Mexico), and thymol (Favela Pro, Sinaloa, Mexico) were used for the synthesis of hydroxysalts. Glycerol (Favela Pro, Sinaloa, Mexico) and CaCl_2_ (Analytyka, Santa Monica, CA, USA) were used for the coatings.

### 2.2. Zinc Hydroxide Salt and Hybrid Synthesis

Zinc layered hydroxide (ZnHSL) and hybrids with thymol (ZnHSL-T) were synthesized following the method described by Velázquez-Carriles et al. [31]. The structure was corroborated with XRD (PANALYTICAL, Malvern, UK) in a 2θ angle from 10–70, a step of 0.02, and a collection time of 30 s, while the micrographies were collected with a SEM microscopy (FE-SEM TESCAN Mira3, Tempe, AZ, USA) at 15 kV.

### 2.3. Filmogenic Solutions

**Figure 1:**
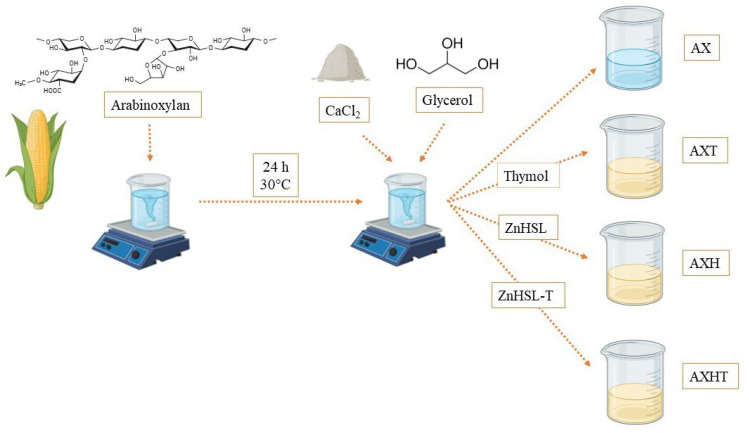
Filmogenic solution preparation.

Edible coatings were prepared according to Shivangi et al. [34]. For the base solution, 3 g of arabinoxylan were diluted in 100 mL of distilled water with constant stirring for 24 h at room temperature (30 °C). Then, glycerol was added in a 0.5% proportion and 10 mg of CaCl_2_ as a cross-linking agent [35]. This base filmogenic solution was labeled as AX. Finally, thymol, ZnHSL, and ZnHSL-T were added at 1% of the total solution; each combination was labeled AXT, AXH, and AXHT, respectively. All mixtures were stirred for 30 min at room temperature, and a portion was poured into Petri dishes and dried in a convection oven for characterization (casting method); the remaining solution was kept at 4 °C until use. A scheme of the procedure is depicted in Figure 1.

### 2.4. Filmogenic Solution and Edible Coating Characterization

Filmogenic solutions were analyzed in an FT-IR spectrophotometer (Nicolet iS5, ThermoScientific, Waltham, MA, USA) from 4000 to 400 cm^−1^ with 32 scans of resolution. Viscosity was measured by adding 45 mL of solutions in a viscosimeter (BROOKFIELD LVT 299301, Middleboro, MA, USA); turbidity was determined in a turbidimeter (Hangzhou Qiwei Instrument ZD-10A, Zhejiang, China), while total soluble solids were quantified in a refractometer (SOONDA, Shanghai, China).

For edible coatings prepared with the casting method, color was measured with a colorimeter (3nh SC-10, Guangdong, China) where values for a, b, and L were recorded and used to calculate total differential color (*ΔE*), Chroma (C*), and Hue angle (*H*°), with Equations (1)–(3), respectively [36]. The thickness of the coatings was measured with a digital micrometer (WEN 10725, West Dundee, IL, USA); transparency was determined with the method described by Han and Floros [37] with modifications. Solutions were read at 530 nm in a transmittance reader (VINCKOLOR TH-200, Shenzhen, China), and the results were expressed in the percentage of transmittance (T%) estimated with Equation (4). The moisture content was obtained in a thermobalance (Adam Equipment PMB 202, Milton Keynes, UK); briefly, 2 g of coatings were placed on the metallic plate, and measurements were taken after 33 min.

(1) $$\Delta E^{*}=\sqrt{{\left(\Delta L\right)}^{2}+{\left(\Delta a\right)}^{2}+{\left(\Delta b\right)}^{2}}\;\;\;$$

(2) $$C^{*}=\sqrt{a^{2}+b^{2}}$$

(3) $$H{}^{\circ}=\arctan\left(\frac{b}{a}\right)$$

(4) $$T\left(\%\right)=\log\left(\frac{T_{530}}{n}\right)$$

where *ΔL* is the difference in luminosity, *Δa* is the difference from green to red, *Δb* is the difference from blue to yellow, and *n* is the thickness of the coating.

### 2.5. Antioxidant Activity of Filmogenic Solutions

The antioxidant activity of filmogenic solutions was assessed by inhibition of the 2,2′-azinobis-(3-ethylbenzothiazoline-6-sulfonic acid) (ABTS) radical, following the methodology of Li et al. [38] ABTS radical at 7.5 mM was adjusted to an absorbance of 0.7 at 750 nm, then, 280 µL of the solution and 20 µL of each filmogenic solution (AX, AXT, AXH, AXHT) were added to a 96-well microtiter plate and incubated for 10 min in the dark. Measurements were read at 750 nm in a plate reader (BIORAD, i-Mark, Berkeley, CA, USA), and the results were expressed as a percentage of inhibition (Equation (5)). ABTS radical with methanol was used as a control.

(5) $$ABTS\;inhibition\;\left(\%\right)=\frac{A^{0}+A^{1}}{A^{0}}*100$$

where *A*^0^ is the absorbance of control, and *A*^1^ is the sample absorbance.

### 2.6. Antibacterial Activity of Coatings

To test the antibacterial effect of the edible coatings, *Salmonella enterica* serotypes (Enteritidis, Typhimurium, and Montevideo) donated by the Molecular Biology Laboratory of Universidad de Guadalajara, CUCEI, were evaluated. Bacterial strains were cultured in tryptic soy broth (TSB) and incubated for 24 h at 36 °C before the test, then the cellular density was adjusted to 1 × 10^5^ cell/mL with optical density at 600 nm in a UV-Vis spectrophotometer (Optizen POP BIO, Daedeok, Republic of Korea).

Cherry tomatoes (*Solanum lycopersicum* var. *cerasiforme*) at maturity stage 6 (deep red, with a red color exceeding 90%) [39], similar in size, color, and texture, were purchased in local markets of Guadalajara, Mexico. Fruits were washed and disinfected in sodium hypochlorite (20 mg/L) for 20 s, washed again to eliminate chlorine residue, and dried at room temperature (30 °C). Filmogenic solutions of each treatment were prepared, and tomatoes were coated in two dipping cycles of 30 s and allowed to dry at room temperature (30 °C) [40], followed by inoculation with the *Salmonella* suspensions previously prepared by immersion. Tomatoes were maintained at room temperature (30 °C) for 12 days and sampled for *Salmonella* recovery at different times (1, 3, 6, 9, 12 days). Tomatoes without any coating were used as a control [41].

For *Salmonella* recovery, tomatoes were submerged in buffered peptone water (BPW) and agitated manually for 1 min to detach the cells. Serial dilutions were made in BPW, cultured by plate extension over Xylose Lysine Desoxycholate (XLD) Agar, and incubated for 24 h at 36 °C. The colony forming units (CFU) were counted, and the results were expressed as a percentage of the initial count estimated with Equation (6) [42].

(6) $$Bacterial\;survival\;\left(\%\right)=\frac{C_{n}}{C_{0}}*100$$

where *C_n_* is the count at each sampling day, and *C*_0_ is the initial count.

### 2.7. Physicochemical Characteristics of Coated Tomatoes

Tomatoes were coated as described previously and maintained at room temperature (30 °C) for 12 days, with evaluations at different times (1, 3, 6, 9, 12 days). Each tomato was weighed at the beginning of the test, and weight loss was determined using an analytical balance. Weight loss was calculated as described in Equation (7).

(7) $$Weight\;loss\;\left(\%\right)=\frac{m_{0}-m_{n}}{m_{0}}*100\;$$

where *m*_0_ and *m_n_* are the weight of the tomato at day 0 and sampling day, respectively. Color parameters (*ΔE*, C*, and *H*°) were determined with Equations (1)–(3), while lycopene content was calculated with Equation (8) and expressed as mg of lycopene per 100 g [43].

(8) $$Lycopene=11.848*\frac{a}{b}+1.5471$$

pH of the tomatoes was measured with a potentiometer (HANNA H06310595, Cluj-Napoca, Romania); total soluble solids (TSS) were determined in a refractometer SOONDA. Finally, for titratable acidity, a tomato solution was prepared in distilled water (1:10), and phenolphthalein (0.1 N) was used as an indicator. The solution was titrated with NaOH (0.1 M), and the volume consumed was used to estimate acidity with Equation (9).

(9) $$Acidity\;\left(\%\right)=\frac{N*VD*M_{eq}}{M}*100\;$$

where *N* is the normality of NaOH, *VD* is the volume of NaOH consumed, *M_eq_* are milliequivalents of acid, and *M* is the sample size (g).

### 2.8. Statistical Analysis

All experiments were performed in triplicate and analyzed using simple ANOVA, followed by the Tukey test. A statistical difference was considered when *p* < 0.05, and the analysis was conducted using Statgraphics Centurion XVIII (V. 19.5.01).

## 3. Results and Discussion

### 3.1. Nanomaterials Characterization

**Figure 2:**
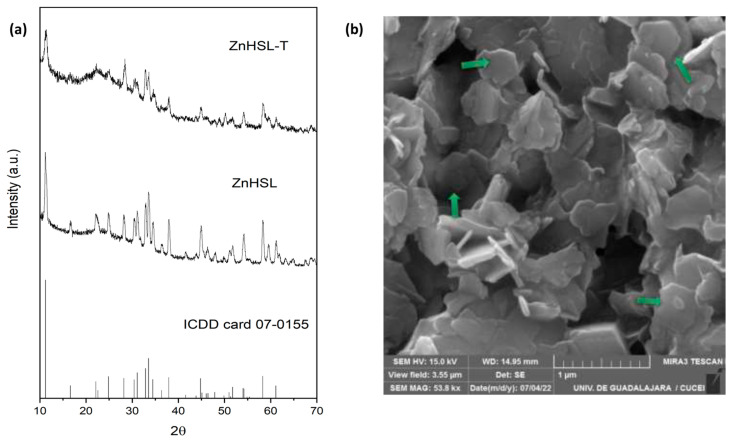
Zinc hydroxide salt and hybrid characterization. (**a**) XRD diffractograms; (**b**) SEM.

Figure 2 shows the characterization of zinc layered hydroxide salt (ZnHSL) and its hybrid with thymol (ZnHSL-T). The XRD diffractogram depicts the characteristic signals of Simonkolleite (ICDD card 07-0155), corroborating the formation of the layered compound. At the same time, the hybrid maintained the signals with reduced crystallinity, as evidenced by the reduction in intensity and widening of peaks, which can be attributed to interaction with thymol (Figure 2a) [32]. The morphology in the SEM images reveals a typical hexagonal structure (green arrows) characteristic of layered hydroxides; the lateral dimension, estimated using ImageJ software (V. 1.54p), was approximately 0.331 µm (Figure 2b). A similar result was previously observed, where the intensity of signals in the XRD diffractograms decreased when a hydroxide salt was in contact with biomolecules such as thymol [31].

### 3.2. Characterization of Filmogenic Solution and Edible Coating

#### 3.2.1. Fourier Transform Infrared Spectroscopy

**Figure 3:**
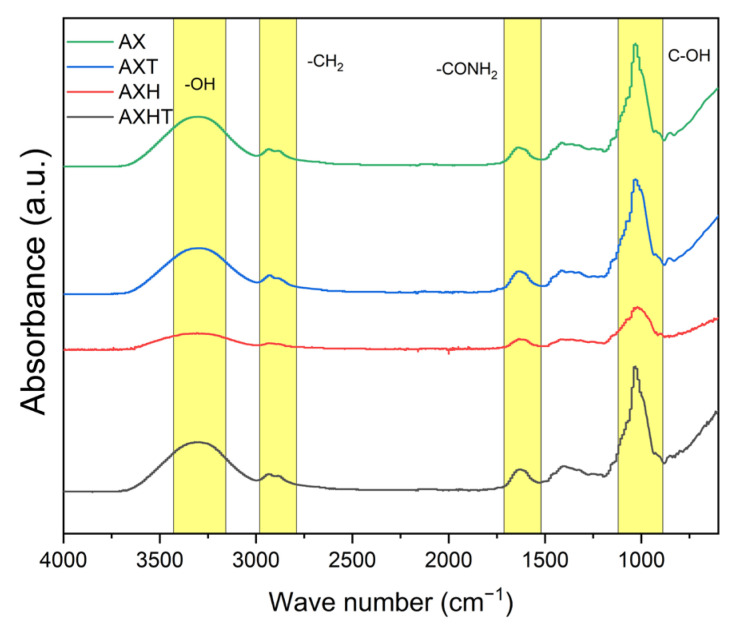
Filmogenic solutions FTIR spectra.

The FTIR spectra of filmogenic solutions (AX, AXT, AXH, and AXHT) are depicted in Figure 3. Signals between 3500 and 3100 cm^−1^ can be associated with the vibration of -OH groups, while C-OH and C-O-C glycosidic bonds are observed around 1000 cm^−1^ [44,45,46]. Amide I and amide II can be observed in the region near 1600 cm^−1^, which is related to proteins. Signals at approximately 2900 cm^−1^ correspond to CH_2_ groups [47]. AXT and AXHT have similar peaks and intensities to AX, suggesting a possible incorporation of thymol and the hybrid into the polymeric net due to the hydrophobic amino acids and lipids of the arabinoxylan [48,49], where the structure is sustained by Van der Waals interactions. On the other hand, AXH exhibits the same signals with lower intensities; this decrease can be attributed to the lesser entrapment of hydroxides, as they lack hydrophobic behavior, in contrast to when thymol is present. Additionally, a probable anion-interchange may be occurring with smaller molecules characteristic of the formulation [50].

#### 3.2.2. Physicochemical Properties of Filmogenic Solutions

Table 1 shows the results for viscosity, turbidity, and total soluble solids (TSS) of filmogenic solutions. These parameters are crucial for adhesion, substrate absorption, thickness, and coating uniformity when applied to foods [51]. No significant differences were found in TSS, possibly due to the insertion of thymol and hydroxide salts between the polysaccharides. Viscosity also showed no significant difference, although it is interesting that in the presence of thymol, the average value was lower (10 cp) than when no molecules were added or when zinc hydroxide was used without thymol. This reduction in viscosity magnitude has been observed in other polysaccharides, such as potato starch with thymol, as the assembly and increase of negative charges on the surface may lead to micelle formation, thereby reducing the particle size and, consequently, the viscosity of the solution [52]. Regarding turbidity, an increase in NTU is observed when different components are included, with AXHT exhibiting the highest turbidity values. The increase in NTU could be due to the rising molecular weight of the system [53], where not only arabinoxylan is participating, but also the zinc hydroxide salt and thymol.

The color parameters of the coatings prepared with the casting method are depicted in Table 2. For all coatings, a tendency to be lightly red (positive value for a*) was observed with no significant difference, while yellow tones increased when ZnHSL and ZnHSL-T were included in the formulation (positive value for b*); also, luminosity (L*) was higher when ZnHSL-T was added compared to other formulations, which impacted the total differential color (ΔE*) as well. The Chroma value (C*) was slightly different for AXH and statistically different for AXHT with the control AX, but the Hue angle (H°) exhibited no significant differences. The color of the ZnHSL-T powder was light yellow, and thus, b* increased significantly when it was introduced to the coating. Weng et al. [54] prepared a coating of arabinoxylan at 2%, where values were different compared to the results obtained in this study, which could be attributed to the polymer extraction method.

Regarding the thickness, transparency, and moisture content of edible coatings (Table 2), it can be seen that the inclusion of ZnHSL and ZnHSL-T significantly reduced the length and transparency of the coatings. The moisture content was not modified with nanomaterials or thymol alone. It has been demonstrated that the concentration of biopolymers has a direct impact on the thickness of coatings, as reported by Alzarea et al. [55], who used a 3% solution of arabinoxylan and 2% sodium alginate for their coatings, obtaining a thickness of 0.276 mm. The type of cross-linker may affect the net formation, and thus the thickness and transparency may change. The thickness of the coatings also modifies the transparency, which could be related to the arrangement of the molecules [56,57]. In AXHT formulation, transparency was the highest, corresponding to the L* parameter.

The inclusion of zinc, silver, and titanium nanoparticles in chitosan-based coatings demonstrates that higher concentrations of particles result in modifications to color and transparency, with a corresponding reduction in transparency as the concentration increases. Additionally, the authors incorporated grape extracts, which not only altered the color but also the aroma of the coatings [16]. These parameters can be related when including compounds such as thymol, as they can affect sensory properties that may lead to consumer rejection of the product [58]. The moisture content, on the other hand, was not affected by the modifications to the formulation.

### 3.3. Antioxidant Capacity of Filmogenic Solutions

**Figure 4:**
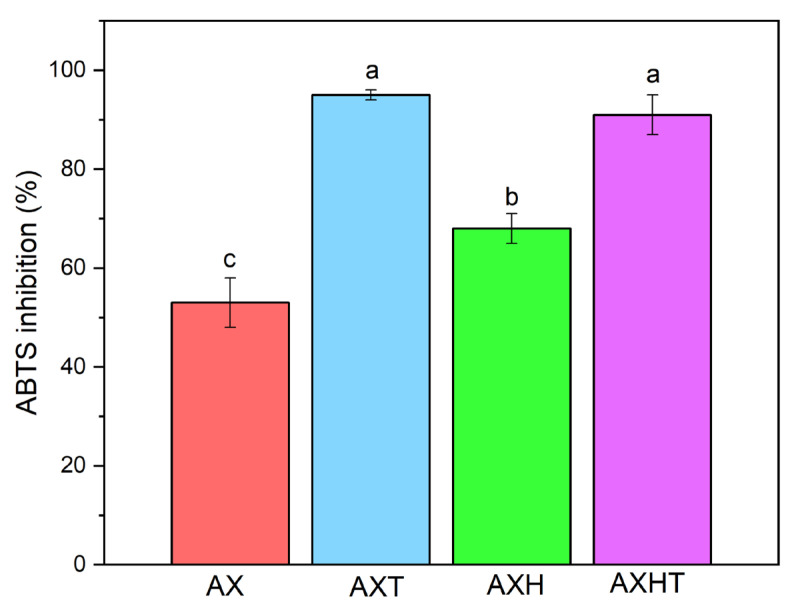
ABTS radical inhibition of arabinoxylan-based filmogenic solutions. Different letters represent significant differences (*p* < 0.05).

Inhibition of radicals, such as ABTS, provides information about hydrophilic and hydrophobic compounds that donate hydrogens for reduction, as evidenced by the loss of blue color in the ABTS solution [38]. Figure 4 depicts inhibition of the ABTS radical with AX, AXT, AXH, and AXHT. Interestingly, ZnHSL-T of AXHT increased the inhibition of the filmogenic solution, and, as expected, being a natural antioxidant, thymol significantly increased the inhibition of the ABTS radical [59]. The hybrid composed of zinc layered hydroxide salt and thymol exhibited a statistically similar percentage of inhibition of ABTS radical as thymol alone, above 90%. However, it should be noted that the concentration of thymol in this formulation was lower than that of free thymol. It has been reported that cross-linked arabinoxylans exhibit higher radical scavenging, chelating capacities, and higher antioxidant activity than compounds alone [53]. The inactivation of free radicals associated with phenolic and reducing sugars in the molecule could delay lipid oxidation in food, thereby extending its shelf life [50].

### 3.4. Efficiency of Edible Coatings on Salmonella Adherence

**Figure 5:**
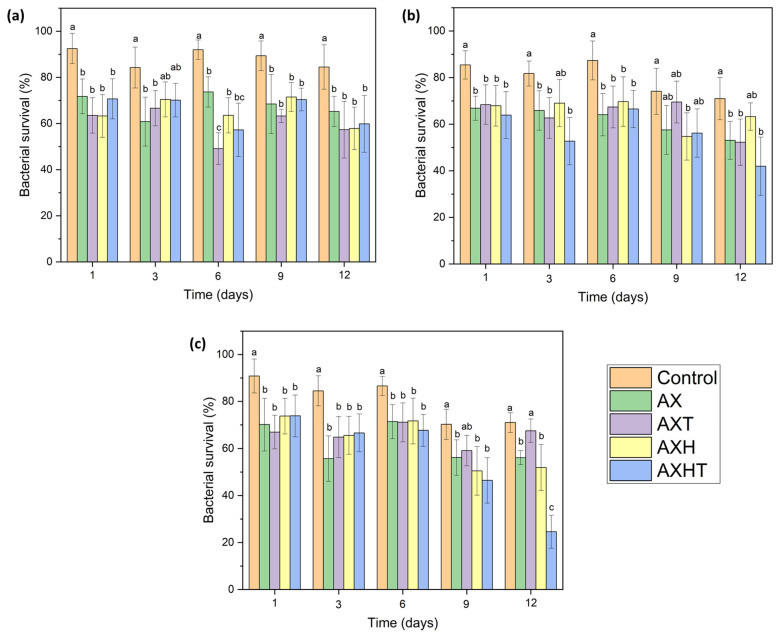
Recovery of survival *Salmonella enterica* from tomatoes coated with edible coatings. (**a**) *Salmonella* Enteritidis; (**b**) *Salmonella* Typhimurium; (**c**) *Salmonella* Montevideo. Different letters represent significant differences (*p* < 0.05).

The efficiency of edible coatings in reducing the survival of *Salmonella* strains on tomato surfaces is depicted in Figure 5. *Salmonella* Enteritidis reduced its population from 90 to 60% with all formulations (Figure 5a), while *Salmonella* Typhimurium had similar results with AX and AXT (Figure 5b); interestingly, AXHT was able to reduce the surviving cells to 40%. Finally, the *Salmonella* Montevideo surviving cell count was significantly reduced by the end of the test on the tomato surface coated with AXHT (24%), resulting in a 50% decrease in population (Figure 5c). All treatments maintained the population of *Salmonella* strains below the control value, possibly due to the antibacterial effect of the coating components, as well as limited spaces for adherence.

Thymol has been reported as an antimicrobial molecule against *Salmonella* since it can increase membrane permeability, provoke leakage of intracellular components, and, consequently, cell death [60]. Layered hydroxide salts are known for the controlled delivery of biomolecules encapsulated within their structure [31,61,62]; this suggests that thymol can be released from the laminar structure in a controlled manner, preventing the reproduction of bacterial cells, or even killing them. Similar results for *S*. Typhimurium were found for broccoli coated with methylcellulose and extracts of rosemary, oregano, and thyme at 4 °C for 12 days [63], while grapefruit seed extract entrapped in chitosan coatings reduced the 2 Log of *Salmonella* spp. in cherries stored at 25 °C [64]. The formulations presented in this research focused on avoiding using energy for storage (refrigeration), which could reduce costs and waste in points of sale. Additionally, it has been reported that these layered materials are non-toxic at the concentration used in this study [62], which suggests that their application is safe for consumption. So far, this is the first time that a hybrid composed of zinc layered hydroxide salt and thymol has been used in an arabinoxylan matrix for edible coating production.

### 3.5. Physicochemical Properties of Coated Tomatoes

**Figure 6:**
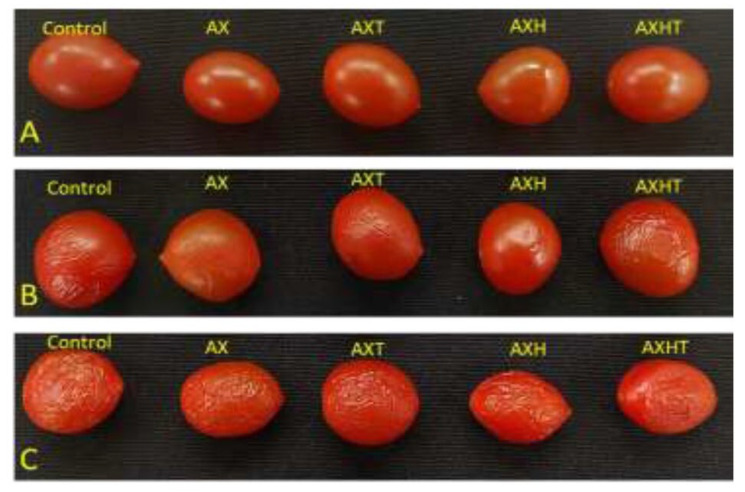
Tomatoes coated with different treatments. (**A**) Day 1; (**B**) day 6; (**C**) day 12.

The physicochemical properties of tomatoes coated with different solutions were analyzed in a 12-day experiment. Figure 6 shows the tomatoes with the different treatments during evaluation at day 1 (Figure 6A), day 6 (Figure 6B), and day 12 (Figure 6C). Interestingly, by the sixth day, the quality of the control began to decrease, evidenced by its turgor loss, while the coated tomatoes reduced this phenomenon. This was also observed at the end of the experiment, where turgor loss was evident in all treatments; however, it was slightly less pronounced in coated tomatoes compared to the control.

#### 3.5.1. Weight Loss

**Figure 7:**
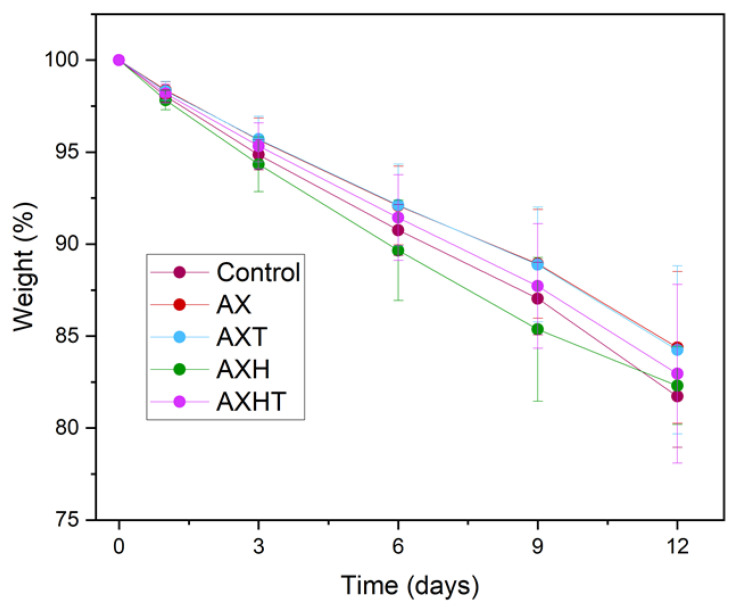
Weight loss of tomato with arabinoxylan-based edible coatings.

To determine weight loss, tomatoes were weighed at day 0 and on the day of sampling (Figure 7). Arabinoxylan edible coated tomatoes exhibited a weight loss of 13%, while control tomatoes lost around 20%, all in a linear behavior. Tomatoes are climacteric fruits with a soft texture that experience decay during storage. The main factor associated with shelf life during postharvest is the rise of the respiratory rate that leads to ripening and decay, which affects physicochemical properties [65,66]. The reduction in physiological weight loss of tomato cherries with edible coatings is primarily due to the formation of a semipermeable barrier that minimizes gas exchange, such as O_2_, CO_2_, and H_2_O, thereby reducing fruit metabolism and, consequently, the respiratory rate, as well as heat and ethylene production [15].

#### 3.5.2. Color Parameters

During the 12-day test, the color of tomatoes coated with the different arabinoxylan formulations was measured for Total color difference (TCD), Chroma, and Hue angle parameters. Table 3 presents the values for each treatment, with TCD compared to the control. Interestingly, each treatment individually exhibited constant values for the three parameters throughout the 12 days; however, the TCD differed from the control, with values around 2. On the other hand, the averages for C* and H° did not show a statistically significant difference (*p* > 0.05).

Results for TCD were found to be distinct throughout the 12-day test for tomatoes coated with each of the arabinoxylan edible coatings. These differences can be attributed to the b* parameter shown in Table 2, where a slight tendency towards a yellow color may modify the TCD of the tomatoes. Modifications in color due to edible coatings on fruits must be considered, as they can affect consumer preferences [16]. An edible coating prepared with arabinoxylan and β-glucan stearic acid (4%) was applied to apples, where no significant changes in color were observed during a 30-day storage period [22].

#### 3.5.3. Lycopene, pH, Soluble Solids, and Titratable Acidity

Table 4 exposes the lycopene content, pH, and soluble solids of tomatoes coated with arabinoxylan formulations. The lycopene content in the control was slightly reduced, from 10.14 to 9.21 mg/100 g, while the AX and AXT coatings maintained a lycopene content between 8.01 and 8.92 mg/100 g. However, no significant difference was observed (*p* > 0.050). On the other hand, AXH and AHXT maintained the content throughout 12 days, with values ranging from 9.01 to 9.87 mg/100 g.

Soluble solids were higher at day 1 in tomatoes with coatings, as thymol and hydroxides were included in the formulations and increased over time, but reached a similar value to the control (9.43–9.53) by the last day of testing. Using coatings without nanoparticles resulted in lower values of total solids in tomatoes stored at 23 °C for 13 days, with values around 4% of TSS [67]. This behavior could be attributed to modifications in the permeability that arabinoxylan coatings may produce on the surface of the tomato, which can reduce O_2_ transit, as well as ethylene production [68]. The pH increased by day 6 and then decreased slightly on the last day, being significantly different for all treatments, which is attributed to the increase in organic acids in the tomato during storage. Similar results were obtained for cherry tomatoes coated with CMC-blackberry anthocyanin extract, stored at 15 °C for 15 days, with no statistical difference compared to uncoated tomatoes [69].

Lycopene is a carotenoid responsible for the red color in tomatoes; it has many pharmacological properties, such as antioxidant, photoprotective, antidiabetic, and antimicrobial [70]. However, this molecule is susceptible to degradation due to oxidation and isomerization, which reduces its antioxidant potential and health benefits [71]. Tomatoes at maturity stage 2 (less than 10% green) coated with carnauba and mineral oil exhibited lycopene content of 10.77 and 11.35 mg/100 g during storage for 28 days at 10 °C, while tomatoes at maturity stage 4 (30–60% different color than green), exhibited values of lycopene content of 14.92 and 15.47 mg/100 g [72]. It should be noted that the results obtained in this study were lower because tomatoes were kept at 30 °C, rather than in refrigeration, where lycopene production typically occurs [39]. Generally, consumers prefer high-quality fruits and vegetables, which are evaluated based on their color, freshness, and taste. Nonetheless, the postharvest supply chain of tomatoes may affect their quality due to poor temperature control during storage [8]. Although no significant difference was found in lycopene content, it is worth noting that the tendency differed between the control and treatments with layered compounds, where the control reduced the content, while AXH and AXHT remained constant. This suggests that the antioxidant activity of coated tomatoes stored at room temperature can be preserved.

#### 3.5.4. Titratable Acidity

Total acidity in foods is often used as a parameter for assessing acid flavor, and it is determined by titratable acidity [73]. Fruit ripening is associated with changes in organic acid production, which reach high values during the fruit’s development. These acids tend to degrade when fruit ripens, favoring the production of sugars responsible for their sweet flavor [74]. All treatments exhibited the same behavior as the control, with results statistically similar (*p* < 0.05). In Table 5, the average of titratable acidity (TA) for all treatments per day is depicted, where the TA increased at day 3 and remained constant towards the end of the experiment. In a study by Taşdelen and Bayindirli [67], an edible coating made of chitosan was applied to tomatoes and stored for 12 days, resulting in a total acidity (TA) percentage of 0.3% at the end of the test. In comparison, the coat formulation with arabinoxylan and nanoparticles containing thymol in this study exhibited higher acidity values, thereby preserving acidic compounds from degradation. In other studies using CMC-blackberry anthocyanin extract as a coating for tomatoes, the total acidic content was reduced over a 12-day period, exhibiting values lower than 0.5 g citric acid/100 g [69]. This behavior of the TA can be related to the pH measured in the previous section. According to Ruelas-Chacon et al. [75], organic acids form as the fruit ripens, and upon full ripeness, these acids degrade.

## 4. Conclusions

An edible coating composed of arabinoxylan from maize waste and hybrids of layered hydroxide salts with thymol (AXHT) was used for the first time on tomato cherry to prevent *Salmonella* spp. adherence. The edible coatings containing thymol exhibited high antioxidant activity by inhibiting the ABTS radical, indicating that the bioactive molecule was effectively incorporated into the zinc layered compound. Lycopene content was reduced throughout the experiment in the control tomato. In contrast, the AXHT edible coating maintained its integrity for 12 days at room temperature, suggesting a delay in the degradation of this molecule. On the other hand, the color of the fruits was less modified than the other formulations, where AXH and AXHT exhibited values similar to those of the control. Additionally, other physicochemical properties, such as pH, soluble solids, and titratable acidity, were not significantly altered, which contributes to the preservation of the characteristics of tomatoes. Regarding the adherence of *Salmonella* spp., AXHT reduced bacterial count significantly higher than the formulations with thymol or zinc layered compound alone, possibly attributed to the reduction of sites of adhesion and the antimicrobial activity of thymol. The *Salmonella* Typhimurium population was the most sensitive serovar, showing a 50% reduction in AXHT, which suggests that this formulation can be used to reduce cross-contamination.

The results obtained in this research provide valuable information that can revolutionize the postharvest process, delivering safer and more nutritious food to consumers without requiring energy-intensive technologies, such as refrigeration, to maintain the suitability of fruits for consumption in terms of bioactive compounds like lycopene.

## Figures and Tables

**Table 1:** Total soluble solids, viscosity, and turbidity of arabinoxylan filmogenic solutions.

Filmogenic Formulation	TSS (°Brix)	Viscosity (cP)	Turbidity (NTU)
AX	3.2 ± 0.5 ^a^	20.0 ± 10.0 ^a^	1220 ± 144 ^a^
AXT	3.3 ± 0.2 ^a^	10.0 ± 0.0 ^a^	1376 ± 51 ^a^
AXH	3.3 ± 0.3 ^a^	20.0 ± 10.0 ^a^	2233 ± 124 ^b^
AXHT	3.3 ± 0.3 ^a^	10.0 ± 0.0 ^a^	3613 ± 65 ^c^

Different letters represent significant differences (*p* < 0.05).

**Table 2:** Color, thickness, transparency, and humidity of arabinoxylan-based edible coatings.

Parameter	Edible Coating Formulation			
	AX	AXT	AXH	AXHT
L*	37.57 ± 1.14 ^a^	38.30 ± 2.59 ^a^	39.56 ± 0.11 ^a^	47.35 ± 0.60 ^b^
a*	4.04 ± 0.71 ^a^	4.19 ± 0.86 ^a^	4.05 ± 0.78 ^a^	4.33 ± 1.23 ^a^
b*	15.05 ± 0.03 ^a^	14.42 ± 1.14 ^a^	18.19 ± 2.68 ^ab^	21.32 ± 3.78 ^b^
ΔE*	NA	2.99 ± 0.66 ^a^	2.79 ± 2.31 ^a^	10.52 ± 1.57 ^b^
C*	15.69 ± 0.47 ^a^	15.03 ± 1.32 ^a^	18.67 ± 2.42 ^ab^	21.82 ± 3.40 ^b^
H°	73.76 ± 2.22 ^a^	73.90 ± 2.00 ^a^	77.09 ± 4.00 ^a^	77.92 ± 5.75 ^a^
Thickness (mm)	0.199 ± 0.053 ^a^	0.196 ± 0.029 ^a^	0.152 ± 0.022 ^b^	0.163 ± 0.018 ^b^
Transparency (%)	2.343 ± 0.015 ^a^	2.307 ± 0.052 ^a^	1.834 ± 0.187 ^b^	1.681 ± 0.138 ^b^
Moisture (%)	15.5 ± 0.2 ^a^	15.0 ± 0.3 ^a^	15.0 ± 0.1 ^a^	15.0 ± 0.4 ^a^

Different letters represent significant differences (*p* < 0.05).

**Table 3:** Average of color parameters of tomatoes with arabinoxylan coatings at room temperature for 12 days.

Parameter	Control	AX	AXT	AXH	AXHT
TCD		2.02 ± 1.45	2.14 ± 1.34	1.84 ± 0.76	1.85 ± 1.23
C*	18.71 ± 1.16	17.80 ± 1.09	17.62 ± 1.00	18.43 ± 1.22	18.55 ± 1.10
H°	55.69 ± 2.36	59.38 ± 2.84	58.54 ± 2.90	55.73 ± 2.87	55.87 ± 2.35

TCD is classified as: Very distinct (TCD > 3); distinct (1.5 < TCD < 3); Slightly distinct (TCD < 1.5).

**Table 4:** Lycopene content, pH, and soluble solids of tomatoes with edible coatings formulations.

Parameter	Day	Control	AX	AXT	AXH	AXHT
Lycopene content (mg/100 g)	1	10.14 ± 0.14 ^aA^	8.92 ± 0.67 ^aB^	8.15 ± 0.76 ^aB^	9.15 ± 0.64 ^aB^	9.68 ± 0.50 ^aAB^
	3	10.01 ± 1.10 ^abA^	9.25 ± 0.93 ^aA^	9.16 ± 0.49 ^aA^	9.44 ± 0.64 ^aA^	8.93 ± 0.37 ^aAB^
	6	9.23 ± 0.53 ^bA^	8.26 ± 0.83 ^aAB^	8.59 ± 0.02 ^aB^	9.86 ± 0.65 ^aA^	9.83 ± 0.96 ^aA^
	9	9.21 ± 0.68 ^bA^	8.46 ± 0.63 ^aAB^	9.24 ± 0.63 ^aA^	9.21 ± 1.21 ^aA^	9.64 ± 1.09 ^aAB^
	12	9.66 ± 0.71 ^abA^	8.01 ± 0.77 ^aB^	8.44 ± 1.70 ^aAB^	9.87 ± 1.04 ^aAB^	9.91 ± 0.48 ^aA^
pH	1	4.44 ± 0.14 ^b^	4.42 ± 0.21 ^a^	4.31 ± 0.11 ^ab^	4.41 ± 0.02 ^c^	4.21 ± 0.18 ^b^
	3	4.61 ± 0.07 ^ab^	4.54 ± 0.19 ^a^	4.40 ± 0.14 ^a^	4.42 ± 0.34 ^bc^	4.54 ± 0.21 ^ab^
	6	4.70 ± 0.09 ^a^	4.62 ± 0.12 ^a^	4.57 ± 0.17 ^a^	4.58 ± 0.15 ^ab^	4.61 ± 0.15 ^ab^
	9	4.63 ± 0.08 ^ab^	4.58 ± 0.19 ^a^	4.58 ± 0.04 ^a^	4.72 ± 0.07 ^a^	4.39 ± 0.14 ^b^
	12	4.63 ± 0.08 ^ab^	4.56 ± 0.01 ^a^	4.49 ± 0.14 ^a^	4.61 ± 0.03 ^b^	4.63 ± 0.03 ^a^
Soluble solids (°Brix)	1	6.57 ± 0.67 ^cB^	7.77 ± 0.55 ^bA^	7.30 ± 1.18 ^bcA^	7.73 ± 0.50 ^abA^	7.87 ± 0.12 ^bA^
	3	8.37 ± 1.08 ^bAB^	8.30 ± 1.08 ^abAB^	7.40 ± 0.26 ^cB^	8.20 ± 2.25 ^aAB^	7.10 ± 1.06 ^bB^
	6	8.70 ± 0.20 ^bAB^	8.03 ± 1.37 ^abAB^	9.10 ± 0.20 ^aA^	7.25 ± 0.55 ^abB^	8.30 ± 1.28 ^abAB^
	9	9.67 ± 0.15 ^aAB^	9.30 ± 0.20 ^aB^	8.93 ± 0.75 ^abBC^	9.98 ± 1.56 ^aA^	8.43 ± 0.85 ^abC^
	12	9.43 ± 1.54 ^abAB^	8.07 ± 0.58 ^bB^	8.17 ± 0.25 ^bB^	8.77 ± 2.29 ^aAB^	9.53 ± 0.70 ^aA^

Lowercase letters indicate statistical differences in columns (*p* < 0.05); uppercase letters indicate statistical differences between rows (*p* < 0.05).

**Table 5:** Titratable acidity of uncoated and coated tomatoes with arabinoxylan formulations.

Day					
0	1	3	6	9	12
0.36 ± 0.08 ^a^	0.32 ± 0.06 ^a^	0.66 ± 0.23 ^b^	0.72 ± 0.22 ^b^	0.70 ± 0.17 ^b^	0.62 ± 0.17 ^b^

Values represent the average of the control and treatment groups per day; uppercase letters indicate statistical differences between rows (*p* < 0.05).

## Data Availability

The original contributions presented in this study are included in the article. Further inquiries can be directed to the corresponding authors.

## Abbreviations

The following abbreviations are used in this manuscript:

AX	Arabinoxylan edible coat
AXT	Arabinoxylan edible coat with thymol
AXH	Arabinoxylan edible coat with zinc hydroxide salt
AXHT	Arabinoxylan edible coat with hybrid of zinc hydroxide and thymol
ZnHSL	Zinc layered hydroxide salt
ZnHSL-T	Hybrid of zinc layered hydroxide and thymol
TCD	Total color difference
TSS	Total soluble solids
TA	Titratable acidity
